# Plasma Neutrophil Gelatinase-Associated Lipocalin Is Primarily Related to Inflammation during Sepsis: A Translational Approach

**DOI:** 10.1371/journal.pone.0124429

**Published:** 2015-04-20

**Authors:** Gordon P. Otto, Jorge Hurtado-Oliveros, Ha-Yeun Chung, Kristin Knoll, Thomas Neumann, Hans J. Müller, Marco Herbsleb, Matthias Kohl, Martin Busch, Maik Sossdorf, Ralf A. Claus

**Affiliations:** 1 Center for Sepsis Control and Care, Jena University Hospital, Jena, Germany; 2 Clinic for Anesthesiology and Intensive Care, Jena University Hospital, Jena, Germany; 3 Clinic for Internal Medicine (KIM III)—Rheumatology, Jena University Hospital, Jena, Germany; 4 Department of Sports Medicine and Health Promotion, Friedrich-Schiller-University, Jena, Germany; 5 Department of Medical and Life Sciences, Furtwangen University, Villingen-Schwenningen, Germany; 6 Clinic for Internal Medicine (KIM III)—Nephrology, Jena University Hospital, Jena, Germany

## Abstract

Acute kidney injury (AKI) during sepsis is common and underestimated. Plasma neutrophil gelatinase-associated lipocalin (plasma-NGAL) is discussed as new biomarker for AKI diagnosis, but during inflammation its function and diagnostic impact remain unclear. The association between plasma-NGAL and inflammatory markers in septic patients, but also in healthy controls and patients with chronic inflammation before and after either maximum exercise test or treatment with an anti-TNF therapy were investigated. *In-vitro* blood stimulations with IL-6, lipopolysaccharide, NGAL or its combinations were performed to investigate cause-effect-relationship. Plasma-NGAL levels were stronger associated with inflammation markers including IL-6 (Sepsis: r=0.785 *P*<0.001; chronic inflammation after anti-TNF: r=0.558 *P*<0.001), IL-8 (Sepsis: r=0.714 *P*<0.004; healthy controls after exercise r=0.786 *P*<0.028; chronic inflammation before anti-TNF: r=0.429 *P*<0.041) and IL-10 (healthy controls before exercise: r=0.791 *P*<0.028) than with kidney injury or function. Correlation to kidney injury or function was found only in septic patients (for creatinine: r= 0.906 *P*<0.001; for eGFR: r= -0.686 *P*=0.005) and in patients with rheumatic disease after anti-TNF therapy (for creatinine: r= 0.466 *P*<0.025). In stimulation assays with IL-6 and lipopolysaccharide plasma-NGAL was increased. Co-stimulation of lipopolysaccharide with plasma-NGAL decreased cellular injury (*P*<0.05) and in trend IL-10 levels (*P*=0.057). Septic mice demonstrated a significantly improved survival rate after NGAL treatment (*P*<0.01). Plasma-NGAL seams to be strongly involved in inflammation. For clinical relevance, it might not only be useful for AKI detection during severe inflammation - indeed it has to be interpreted carefully within this setting - but additionally might offer therapeutic potential.

## Introduction

Acute kidney injury (AKI) is a common, most often early event in critical ill patients and it is linked to progression towards chronic kidney disease [1]. Therefore, new biomarkers for the early detection of AKI are under evaluation. Hereby, plasma-NGAL has been identified as one of the most promising candidates for diagnosis and monitoring of AKI [2, 3].

NGAL is a 25 kDa protein of the lipocalin family, which was initially identified in neutrophils, but is also found increased in other tissues in response to various conditions such as ischaemia and infection [4–6]. NGAL itself is stored e.g. in neutrophils within specific granules [7] and is constitutively expressed in several organs including the kidneys [5]. As an iron scavenger, it binds bacterial siderophores, reducing available iron, thereby inhibiting bacterial growth and controlling intracellular iron concentrations [8, 9].

Several data indicate a relationship between plasma-NGAL and immune response [10–12]. Cell culture studies demonstrated an increased NGAL expression after lipopolysaccharide (LPS) [13], but also tumor necrosis factor (TNF) alpha [14] stimulation. Lipocalin-2 deficient mice demonstrated a higher susceptibility for bacterial infections, and in wild type animals undergoing sepsis, a strong correlation between plasma-NGAL, interleukin 6 (IL-6) and interleukin 10 (IL-10), but also with TNF alpha was found [15, 16], which raised the question whether plasma-NGAL might be stronger related to inflammation than to AKI itself. The precise role of plasma-NGAL as marker or mediator in non-infectious/non-inflammatory compared to infectious/inflammatory-related types of AKI is currently unclear and controversially discussed [17–20].

In the present study we investigated the relationship of plasma-NGAL with prototypic markers of inflammation in different cohorts of patients, representing acute and chronic but also mild to severe inflammation with and without AKI, used *in-vitro* models to investigate cause and effect between plasma-NGAL and inflammation and, for confirmation, analyzed outcome of septic animals treated with recombinant Lipocalin-2.

## Results

### Plasma neutrophil gelatinase-associated lipocalin and inflammation in human disease

**Fig 1:**
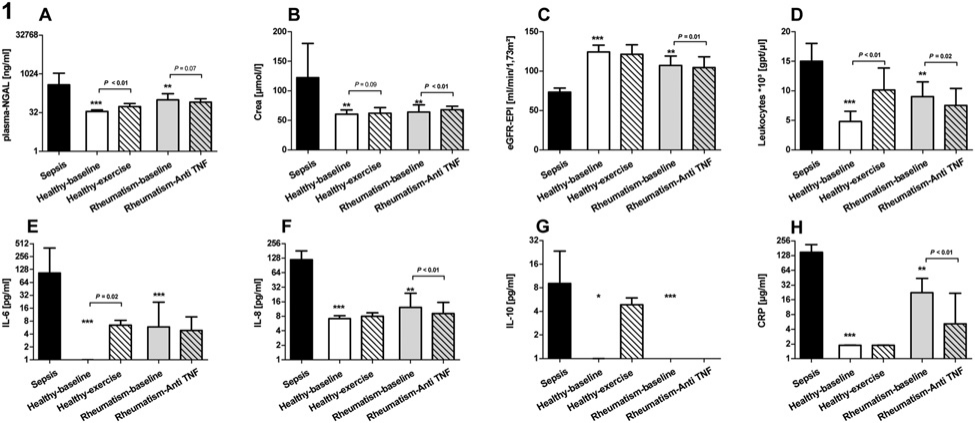
Laboratory finding of patients with sepsis, healthy controls and patients with rheumatic disease. Patients with sepsis (n = 15) are presented with black, healthy controls (n = 8) before inflammatory stimulus by exercise with white open bars and after stimulus with white striped bars and patients with rheumatic disease (n = 23) before treatment with an anti-TNF agent with grey open bars and after treatment with grey striped bars. (A) Plasma-NGAL levels, (B) plasma creatinine levels (Crea), (C) estimated glomerular filtration rate (eGFR-EPI), (D) leukocyte count in whole blood, (E) plasma interleukin 6 levels (IL-6), (F) plasma interleukin 8 levels (IL-8), (G) plasma interleukin 10 levels (IL-10) and (H) plasma levels of C-reactive protein (CRP) are presented. * *P*<0.05, ** *P*<0.01, *** *P*<0.001 compared to sepsis or indicated by clamp to corresponding group. Cytokine detection limits were 2.5 pg/ml for IL-6, 3.6 pg/ml for IL-8 and 3.3 pg/ml for IL-10. Bars and error bars represent median values and interquartile range.

In 15 patients fulfilling the criteria for severe sepsis, we measured plasma-NGAL, plasma Crea and eGFR, CRP, leukocyte count as well as IL-6, IL-8 and IL-10. All these parameters were significantly altered in patients compared to eight healthy controls (*P*<0.01; Fig 1A, 1B and 1C; Table 1 and S1 Table). Within these septic patients, we detected a strong correlation of plasma-NGAL to kidney function indicated by plasma Crea and eGFR as well as with prototypic inflammatory markers including CRP (moderate), PCT (strong), IL-6 (strong) and IL-8 (strong) (*P*<0.02; Table 2). In the group of healthy controls correlations were found for plasma-NGAL only with CRP and IL-10 (strong each, *P*<0.04), but none with plasma Crea or eGFR. All healthy controls showed plasma-NGAL, plasma Crea and eGFR levels within the normal range (Table 1). We speculate that under healthy conditions (without kidney damage and without inflammation) moderate changes in inflammatory status might be predominantly responsible for moderate changes in plasma-NGAL levels. To confirm this hypothesis we evoked a considerable inflammatory stimulus within these healthy individuals by performing a singular maximal running exercise test. We expected that the measure increases markers of inflammation and potentially also plasma Crea levels, released by muscular activity, but not leading to a significant kidney injury. Immediately after exercise a significant increase of plasma-NGAL levels but also in leukocytes count and IL-6 was found (*P*<0.05; Fig 1A, 1B, 1C, 1D and 1E and S1 Table), however, plasma Crea levels were not significantly elevated and within normal ranges. To avoid effects of possible dehydration, parameters were normalized with respect to hematocrit values prior to correlation analyses. Here, plasma-NGAL levels were found to be strongly correlated to IL-8 (*P* = 0.028; Table 2). Following such mild inflammatory stimulus plasma-NGAL levels once more showed no correlation to Crea or eGFR. While in the cohort of septic patients plasma-NGAL correlates to both inflammation and renal markers, in healthy individuals with none to moderate inflammation, but without kidney damage, the observed slight increase in plasma-NGAL might be induced by the inflammatory response.

**Table 1:** Characteristics of patients with sepsis, healthy controls and rheumatic disease.

	Patients with sepsis	Healthy controls	Patients with chronic inflammation	Normal rang
Number of patients, abs.	15	8	23	
Age, years [IQR]	66.00 [49.25–76.25]	27.00 [25.00–28.00]	48.00 [36.00–61.00]	
Gender, male abs. (%)	9 (60.0)	5 (62.5)	11 (47.83)	
Plasma-NGAL, ng/ml [IQR]	381.7 [192.9–1083]	35.00 [29.00–40.62]	98.19 [61.71–172.7]	<100–150
Creatinine, μmol/l [IQR]	122.0 [77.00–180.0]	60.50 [53.25–67.50]	64.00 [58.00–76.00]	Various factors depended <90–110
eGFR, median ml/min/1.73m^2^ [IQR]	73.28 [66.71–78.45]	124.3 [120.7–132.9]	107.3 [95.47–119.2]	Various factors depended >85–116
Leukocyte count, 10^*3*^ gpt/μl [IQR]	15.00 [13.50–18.00]	4.80 [4.25–6.52]	9.00 [6.70–11.50]	Between 4–11
CRP, μg/ml [IQR]	150.0 [67.00–216.00]	1.89 [1.50–1.91]	22.40 [13.30–44.00]	<10
IL-6, pg/ml [IQR]	105.9 [35.33–401.7]	0.00 [0.00–5.33]	5.880 [0.0–22.16]	<1–5
IL-8, pg/ml [IQR]	119.0 [55.25–181.0]	7.18 [1.46–8.21]	12.28 -[9.76–23.84]	<9–10
IL-10, pg/ml [IQR]	9.10 [5.18–23.43]	0.0 [0.0–5.16]	0.0 [0.0–0.0]	<3
ICU mortality, abs. (%)	5 (33.33)			
APACHE II, pts. [IQR]	21.00 [12.00–30.00]			
SAPS II, pts. [IQR]	44.00 [34.00–65.00]			
rel. VO_2_ max, ml/min*kg [IQR]		47.5 [44.9–52.8]		
Lactate max, mmol/l [IQR]		10.8 [9.1–11.3]		
RER max [IQR]		1.16[1.12–1.20]		
DAS28, pts. [IQR]			4.72 [4.18–5.41]	
Active rheumatoid arthritis, abs. (%)			10 (43.48)	
Psoriatic arthritis, abs. (%)			3 (13.04)	
Ankylosing spondylitis, abs. (%)			10 (43.48)	

Selected characteristics of cohorts with different inflammation states and normal ranges are demonstrated. abs absolute numbers, IQR interquartile range, plasma-NGAL plasma neutrophil gelatinase-associated lipocalin, eGFR estimated glomerular filtration rate calculated according eGFR-EPI, CRP C-reactive protein, IL-6 interleukin 6, IL-8 interleukin 8, IL-10 interleukin 10, ICU intensive care unit, APACHE II Acute Physiology And Chronic Health Evaluation II score, SAPS II Simplified Acute Physiology Score II, rel VO2 relative oxygen uptake, RER max maximal respiratory exchange ratio, DAS Disease Activity Score of 28 joints. Data were presented as median values and interquartile range [IQR].

**Table 2:** Correlation of plasma-NGAL with inflammation markers and kidney function.

	Patients with sepsis		Healthy controls				Patients with chronic inflammation			
			before exercise		after exercise^*b*^		before anti-TNF treatment		after anti-TNF treatment	
Marker	r (95% CI)	*P*	r (95% CI)	*P*	r (95% CI)	*P*	r (95% CI)	*P*	r (95% CI)	*P*
Kidney function										
*Creatinine*	**0.906 (0.727–0.970)**	**0.001**	-0.405 (-0.863–0.429)	0.320	0.047 (-0.680–0.728)	0.935	0.115 (-0.324–0.513)	0.600	**0.466 (0.054–0.743)**	**0.025**
*eGFR* ^*a*^	**-0.686 (-0.890–-0.252)**	**0.005**	0.619 (-0.152–0.922)	0.115	-0.238 (-0.807–0.561)	0.5821	-0.170 (-0.553–0.273)	0.439	-0.300 (-0.642–0.140)	0.163
Inflammation										
*Leukocytes*	-0.293 (-0.709–0.274)	0.289	0.262 (-0.543–0.816)	0.536	0.381 (-0.443–0.856)	0.360	**0.526 (0.132–0.776)**	**0.001**	**0.484 (0.077–0.753)**	**0.019**
*CRP*	**0.614 (0.132–0.861)**	**0.015**	**-0.755 (-0.953–-0.106)**	**0.031**	-0.500 (-0.891–0.316)	0.216	**0.426 (0.003–0.719)**	**0.043**	0.399 (-0.029–0.703)	0.059
*PCT*	**0.753 (0.354–0.920)**	**0.002**	n.m.	n.m.	n.m.	n.m.	n.m.	n.m.	n.m.	n.m.
*IL-6*	**0.785 (0.421–0.931)**	**0.001**	-0.518 (-0.896–0.294)	0.197	-0.214 (-0.798–0.578)	0.619	0.162 (-0.280–0.548)	0.459	**0.558 (0.046–0.837)**	**0.031**
*IL-8*	**0.714 (0.280–0.906)**	**0.004**	-0.180 (-0.785–0.601)	0.665	**0.786 (0.182–0.959)**	**0.028**	**0.429 (0.008–0.721)**	**0.041**	0.393 (-0.166–0.761)	0.147
*IL-10*	0.442 (-0.133–0.794)	0.114	**0.791 (0.195–0.960)**	**0.028**	0.311 (-0.504–0.833)	0.462	-0.016 (-0.436–0.410)	0.942	n.c.	n.c.

Correlation of plasma neutrophil gelatinase-associated lipocalin with markers of kidney function or damage and prototypic markers of inflammation in various cohorts representing different states of inflammation.

^a^ calculated according eGFR-EPI,

^*b*^ Values normalized to hematocrit to reduce dehydration effects; n.m. not measured, n.c. not calculated since samples were below detection level. eGFR estimated glomerular filtration rate, CRP C-reactive protein, PCT procalcitonin, IL-6 interleukin 6, IL-8 interleukin 8, IL-10 interleukin 10. Spearman correlation analyses (two tailed) were performed.

To get more details within the continuum between mild (after exercise) and severe inflammation (sepsis), we investigated 23 patients exhibiting active rheumatic diseases with persistent chronic inflammation but no signs of a present AKI (Table 1 and S1 Table). This patient cohort revealed significantly elevated levels of IL-8, CRP, and plasma-NGAL, but also a significant reduction in eGFR as compared to healthy controls (*P*<0.05; Fig 1A, 1F, and 1H). Nevertheless, eGFR in patients with rheumatism was found within the normal range (107.3 ml/min/1.73m^2^ [IQR 95.5–119.2], Table 1). Likewise, plasma Crea levels were not significantly different compared to those in healthy controls, concluding that kidney damage was either absent or evaded confirmation by plasma Crea or eGFR. In contrast to the patients with sepsis, patients with chronic rheumatism presented a significant lower elevation of inflammatory markers such as CRP, leukocyte count, IL-6 and IL-10 (*P*<0.05; Fig 1A, 1B, 1C, 1D, 1E, 1F, 1G and 1H) reflecting the moderate inflammatory condition. Similar to septic patients or healthy controls before and after exercise, moderate correlations of plasma-NGAL to inflammation markers such as IL-8 (as in septic patients and healthy controls after exercise) and CRP (as in septic patients and healthy controls before exercise) were observed (*P*<0.05). Similar to healthy controls, no correlation of plasma-NGAL to plasma Crea or eGFR was found (*P*>0.05; Table 2). Since, the patients with rheumatic disease were older (+ 21 years compared to healthy controls) and presenting with a chronic persistent inflammation, it might be speculated that a correlation of plasma-NGAL with kidney function, which was slightly reduced (as indicated by the decreased eGFR compared to healthy controls), was covered by the inflammatory burden of the underlying disease.

To elucidate whether the increase of plasma-NGAL was rather caused by the chronic inflammation than by any degree of kidney damage, the (same) patients were re-analyzed after their medication was switched—for medical reasons—from conventional disease modifying drugs to an anti-TNF agent. This change in therapy should provoke an improved immune inhibition within this patient cohort. In line to this hypothesis, a significant reduction of IL-8, leukocyte count and CRP levels (*P*<0.05; Fig 1A, 1B, 1C, 1D, 1E, 1F, 1G and 1H and S1 Table) were observed. Plasma-NGAL reduction failed to reach level of significance (*P* = 0.070; Fig 1A). Subsequent to the therapeutic switch it appears that plasma-NGAL levels correlated only moderately with IL-6 and to a minor degree with leukocyte count (*P*<0.040), replacing correlations to CRP and IL-8. Surprisingly, a moderate correlation of plasma-NGAL with Crea was now found (*P* = 0.025). After suppressing inflammation, it might be speculated that the function of plasma-NGAL in terms of a kidney injury marker become obvious. From these human studies we conclude that plasma-NGAL is moderatly to strongly correlated with inflammation and that in a distinct cohort of patients the relationship with the inflammation status might mask the degree of kidney damage. To investigate the interaction between immune response and plasma-NGAL in sterile and infectious inflammation and to elucidate potential cause-effect relationship *in-vitro* whole blood stimulation experiments were performed.

### The impact of plasma-NGAL on inflammation in whole blood *in-vitro*

**Fig 2:**
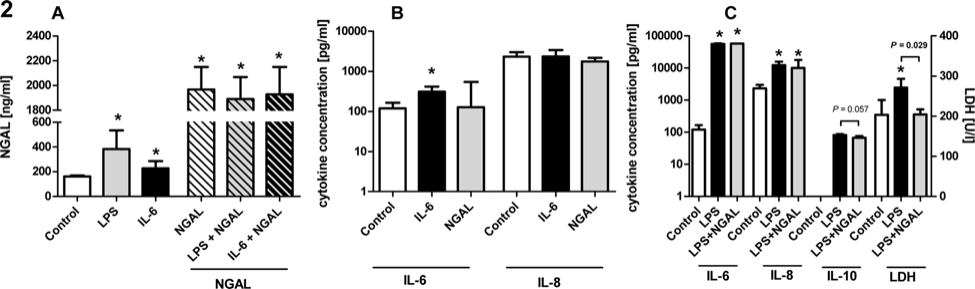
In-vitro stimulation of whole blood. Stimulation experiments with IL-6, LPS, NGAL or their combinations with a total numbers of n = 4 are presented. (A) NGAL levels in vehicle controls (open bar) and after stimulation with LPS (grey filled bar), IL-6 (black filled bar), and after stimulation with only NGAL (open, striped bar), NGAL + LPS (grey, striped bar) and NGAL + IL-6 (black, striped bar). (B) Cytokine levels of IL-6 and IL-8 in control (open bars) and after stimulation with IL-6 (black) or NGAL (grey). (C) Cytokine levels of IL-6, IL-8, IL-10 as well as levels of LDH in control (open bars) and after stimulation with LPS (black) or LPS+NGAL (grey). * *P*<0.05 compared to vehicle control or indicated by clamp to corresponding group. Bars and error bars represent median values and interquartile range.

The cytokine IL-6 is one of the most prominent mediators in septic patients but also in patients with rheumatism. In order to confirm the potential impact of circulating IL-6 for plasma-NGAL levels found in septic patients and patients with rheumatic disease after treatment with an anti-TNF agent, whole blood samples were stimulated with IL-6. After stimulation, a significant increase of plasma-NGAL was found similar to vehicle controls (*P*<0.05; Fig 2A and S2 Table). The stimulation with NGAL did not change the concentration of IL-6 or IL-8 (*P*>0.1; Fig 2B and S2 Table), thus we concluded that NGAL release can be induced by IL-6 but did not change the levels of IL-6 or IL-8 itself.

To mimic an infectious setting as found in septic patients, stimulations using LPS alone or in combination with NGAL were performed. Following LPS, a significant increase of NGAL levels (*P*<0.05; Fig 2A and S2 Table) was observed. The strong increase of NGAL after IL-6 or LPS stimulation was abrogated when blood samples were co-stimulated with NGAL in concentrations as found in septic patients (Fig 2A). Interestingly, when whole blood was stimulated with LPS in combination with NGAL, a trend towards reduced IL-10 levels (*P* = 0.057; Fig 2C and S2 Table) and a maintenance of cellular integrity reflected by a significant reduction of lactate dehydrogenase levels (LDH) compared to stimulation with LPS alone (*P*<0.05; Fig 2C and S2 Table) were found. In combination to LPS, NGAL might mediate reversely its own release and also potentially attenuate the cytokine release of IL-10 and cell toxicity (reduced LDH levels). Since the reduction of the overwhelming inflammatory response and cell damage are substantial therapeutic targets during sepsis these findings might be of relevance [21, 22].

### Plasma neutrophil gelatinase-associated lipocalin as therapeutical target or treatment option during sepsis

**Fig 3:**
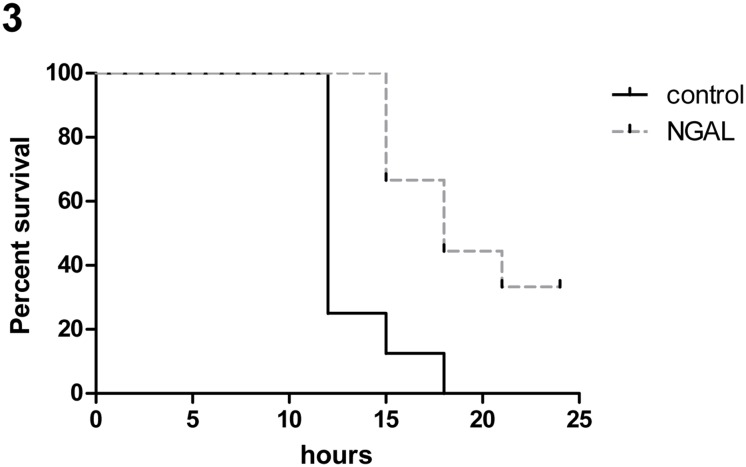
Kaplan Meier survival analyses of mice with sepsis. Kaplan Meier survival analyses of mice with sepsis. Sepsis was induced by peritoneal contamination and infection and subcutaneous treatment with recombinant mouse Lipocalin-2 (n = 9, dotted line in grey) or placebo (n = 8, line in black) was performed. Log-rank test *P*<0.01.

To evaluate whether the potentially beneficial effects of NGAL *in-vivo* have a significant impact on outcome during sepsis, we induced a highly lethal polymicrobial sepsis in mice and treated these with recombinant mouse Lipocalin-2 protein. We decided to use a highly acute model to exclude known bacteriostatic effects of NGAL and to focus on potential effects towards host response. Whereas all mice in the vehicle group died within the first 18 h, 44.4% of animals in the treatment group survived at that time point, displaying a significantly improved survival rate following Lipocalin-2 treatment (*P*<0.01, Fig 3 and S3 Table).

## Discussion

Our data indicate in line to other studies that plasma-NGAL levels are strongly influenced by inflammation, but demonstrate for the first time that plasma-NGAL itself is competent to alter inflammatory response. The data obtained from septic patients confirm results from animal models presenting a correlation of plasma-NGAL with IL-6 during sepsis [15]. Additionally, correlations of plasma-NGAL with multiple inflammatory markers were found in healthy but also disease states and underline the relation between inflammation and plasma-NGAL, not only during infection-triggered inflammation but also in sterile inflammation. Various published studies showed that increased levels of plasma-NGAL can be detected in non-infectious conditions or in patients with normal kidney function [23, 24]. While in patients without any inflammation plasma-NGAL might be a reliable marker to identify a new onset of AKI, in patients with ongoing mild to moderate inflammation the diagnostic value of plasma-NGAL for the detection of AKI might be restricted. In those conditions a minor or moderate increase in NGAL might be more related to inflammation than to AKI itself. In critically ill, septic patients with severe inflammation often suffering from AKI, the increase of plasma-NGAL might be misinterpreted as of exclusively kidney- rather than of combined kidney and inflammatory origin. Since the grade of inflammation is a strong predictor of AKI and renal outcome [25, 26], the predictive value of plasma-NGAL in terms of renal function might be more related to inflammation than to renal damage itself. Especially in patients with severe inflammation e.g. sepsis it is assumed that various factors are responsible for AKI as remote organ injury. During sepsis changes in renal perfusion, beyond other factors overwhelming cytokine release *per se* is involved in progression of kidney damage [27–30]. Since NGAL is an acute phase protein [14], the diagnostic value of plasma-NGAL in terms of being only a marker of kidney damage needs to be interpreted carefully. Nevertheless, it might be helpful for simultaneous monitoring of a potential kidney-damaging inflammation **and** AKI. The potential differences in plasma-NGAL between pure AKI (without infection) and inflammation-related AKI were validated in first clinical studies: Plasma-NGAL was significantly elevated in patients with septic AKI compared to patients with non-septic AKI while renal characteristics were comparable between both groups [31]. As a conclusion NGAL might represent a global biomarker of inflammation. The combination of plasma-NGAL and endotoxin activity assays demonstrated that thereby the detection performance characteristics for identification of septic AKI was improved [32]. Interestingly, these data also show that at least in this setting plasma-NGAL levels strongly overlap between patients with sepsis but without AKI and patients with AKI but without sepsis and therefore might not be useful to distinguish AKI and sepsis.

In addition to clinical data, our study indicates that NGAL is not only increased after cytokine stimulation, but also preserves cellular integrity with the capacity to abrogate cytokine response after stimulation with LPS. In context to the slight but insignificant reduction of IL-10 level after NGAL stimulation, macrophages obtained from NGAL deficient mice displayed a significant increase of IL-10 levels during infection with *Chlamydia pneumoniae* [33]. When investigating the underlying mechanisms it was found that anti-IL-10 treatment reduced ferritin protein levels resulting in reduced iron storage capacity and increased availability of labile iron in the cell [34]. Thus, macrophage iron retention is directly associated with increased IL-10 expression, since IL-10 has previously been shown to increase ferritin expression and cellular iron storage [35]. While anti-IL-10 treatment reduced ferritin protein levels in NGAL deficient macrophages, it increased ferroportin mRNA levels resulting in induction of iron export. The increased expression of IL-10 in NGAL deficient macrophages was be considered to function as a compensatory mechanism to outbalance the high intracellular iron levels by promoting iron storage via incorporating the ion into ferritin rendering this nutrient less available for bacteria [33]. An increase in IL-10 might be a disadvantage for the host in terms of bacterial clearance during acute bacterial infections [36]. After treating macrophages with NGAL neutralizing antibodies or in macrophages from NGAL deficient mice, increased amounts and vitality of bacteria was found [37]. As a consequence, NGAL deficient mice presented a higher susceptibility against mono-bacterial infection with *Escherichia coli* [38].

The final mechanisms of NGAL action are unclear but previous studies have demonstrated that NGAL is protective by inhibiting the formation of High-Mobility-Group-Protein B1 by reducing cytokine production, inflammation [39], and oxidative stress [40]. Additionally it also can prevent AKI itself [41, 42]. In our study the treatment with Lipocalin-2 improved the survival rate of septic animals undergoing severe polymicrobial peritonitis.

Our study has several limitations. The numbers of included patients per investigated cohort were restricted and the results should be revalidated with larger cohorts. Furthermore, the spectrum of measured cytokines was focused onto a small selection, unable to represent the complete network of further known potent inductors of NGAL such as IL-1b [43, 44] and TNF alpha [45], thus these cytokines should be also analyzed. Additionally, the results from the *in-vitro* NGAL stimulation experiments might be biased since a precise differentiation of exogenously administered and endogenously secreted NGAL was not possible. Taking a normal haematocrit into account, the measured supernatant levels indeed represent mostly the levels of NGAL used for stimulation, but might mask minor—potentially relevant changes in endogenous NGAL production. Increasing dosages during NGAL co-stimulation would be helpful to demonstrate a dose-response relationship. Also the combination with siderophores and NGAL should be investigated in more detail. Data from literature show the impact of siderophores and of the iron concentrations, both affecting NGAL function, but these co-factors were not considered in our *in-vitro* experiments. Finally, it was previously reported [46] that the efficacy of anti-inflammatory treatment strategies in sepsis is positively correlated with the severity of the disease and therefore, effectiveness of drugs proposed for treatment of host response should be primarily tested in models with unfavorable outcome. In line with that, we used a severe and highly lethal model to demonstrate a significantly improved survival rate after NGAL treatment, which might over-interpret beneficial results.

In conclusion, our data indicate that the function of circulating plasma-NGAL is not only restricted to the detection of AKI; it might be involved in immune response during inflammation. These findings and the potential therapeutic properties should evoke a reconsideration of our understanding of plasma-NGAL. Plasma-NGAL levels in patients with sepsis have to be interpreted carefully and in context to the inflammation status of the patients.

## Materials and Methods

### Clinical study of patients with sepsis

After obtaining institutional ethical approval (Ethics Commission of the Friedrich-Schiller-University Jena, 2709-12/09) and written informed consent, 15 patients with severe sepsis or septic shock according to the guideline of the American College of Chest Physicians and the Society of Critical Care Medicine [47] were enrolled. This study was performed in the year 2010. Scores and laboratory findings were accessed from the patient data management system. Parameters including age, sex, international classification of disease (ICD-10), length of stay (LOS), ICU mortality rate, the Acute Physiology and Chronic Health Evaluation II (APACHE II) score, the Simplified Acute Physiology (SAPS II) score, plasma levels of creatinine (Crea) and the estimated glomerular filtration rate (eGFR) using the CKD-EPI formula according to Levey et al. [48], C-reactive protein (CRP), procalcitonin (PCT) and leukocyte count were obtained. None of the patient was treated with renal replacement therapy. In total five patients present without AKI, one patient was classified with AKIN 1, seven patients with AKIN 2 and two patients with AKIN 3 stage. Citrated whole blood was centrifuged at 2000xg for 10 minutes at 4°C, plasma was collected and snap-frozen in liquid nitrogen and stored at –80°C until measuring plasma-NGAL and cytokine levels.

### Study of healthy controls before and after inflammatory stimulus

After obtaining institutional ethical approval (Ethics Commission of the Friedrich-Schiller-University Jena, 3433-03/12) and written informed consent, eight young healthy individuals were enrolled. This study was performed in June 2012. Whole blood was obtained pre- and immediately after a maximal incremental exercise test. Exercise was performed by spiroergometry on a treadmill according to a standardized graded protocol (2 km/h-increment, 3-minute stages, starting with a running speed of 6 km/h for females and 8 km/h for males) until volitional exhaustion. Blood lactate concentration and relative oxygen uptake were measured. Plasma was obtained and stored as described above.

### Observational study of patients with rheumatic disease before and after suppressing inflammation

After approval by the local ethic committee (Ethics Commission of the Friedrich-Schiller-University Jena, 3161-06/11) and written informed consent, 23 patients with persistent active rheumatoid arthritis, psoriatic arthritis or ankylosing spondylitis were enrolled. This study was performed from 2012 until 2014. Medical treatment was modified due to underlying diseases from sulfasalazine, methotrexate, prednisolone, or leflunomide to an anti-TNF treatment. The anti-TNF therapy was initiated either with etanercept (n = 12), certolizumab (n = 5), infliximab (n = 1), adalimumab (n = 3) or golimumab (n = 2). Study visits were performed at baseline (before) and up to three months after switch in medication. Data including age, sex, Disease Activity Score of 28 joints (DAS28), plasma levels of creatinine, eGFR, leukocyte count and CRP levels were obtained. Plasma was obtained and stored as described above.

### Whole blood stimulation experiments

For the stimulation experiments hirudinized (50 μg/ml) human whole blood of healthy donors, with no previous history of sepsis or SIRS and undertaking no medications at the time of the study was used. This study was performed in the year 2014. In total, 1 ml aliquots (n = 4) were stimulated for 6 h with LPS (100 ng/ml, from *Escherichia coli* 026:B6, Sigma Aldrich), recombinant human NGAL (1 μg/ml, 10222-H08H, Sino Biological) or IL-6 (500 pg/ml, R&D), or a combination of LPS + NGAL or IL-6 + NGAL in a shaker at 37°C. The used human NGAL preparation in this study was free from siderophores and no additional siderophores or iron were added for these experiments. After stimulation, blood was centrifuged at 2000xg for 10 min at 4°C and plasma aliquots were obtained. Vehicle controls were used as negative control.

### Animal model of sepsis

After approval by the animal welfare committee (Thueringer Landesamt fuer Lebensmittelsicherheit und Verbraucherschutz, 02-046/13) female mice (C57BL/6-background) were randomized to vehicle control (n = 8) and treatment (n = 9) groups and were injected with a highly lethal fecal slurry dose (2 μl/g BW, 80–100% 24-hour mortality) into the right lower quadrant of the abdomen with a 20-gauge cannula [49]. This study was performed in the year 2014. The animals were treated with physiological saline solution (25 μl/g BW) subcutaneously at 1 hour and 13 hours after polymicrobial peritoneal contamination and infection (PCI) induction. In the treatment group recombinant mouse Lipocalin-2 (NGAL) was administered in a single dosage (400 ng/g BW), in combination with saline at 1 hour after PCI induction.

### Measuring NGAL

Plasma-NGAL and NGAL levels from cell culture experiments were measured by commercially available NGAL Rapid ELISA Kit (KIT 037, Bioporto, Denmark) according to the manufacturers instructions. Standards and samples were run in duplicates or triplicates.

### Laboratory measurements as cytokine, lactate dehydrogenase measurements and others

The plasma cytokine concentrations of IL-6, IL-8 and IL-10 were quantified using a flow cytometry-based multiplex assay according to the manufacturers instructions (BD, Cat. No. 551811). Data were acquired by FACS Calibur (BD) and BD CellQuest Pro software. Data were analyzed using the FCAP Array v1.0.1 software. Automated clinical chemistry analyzer (Fuji Dri-Chem 3500i; Sysmex, Germany) was used for measurements of lactate dehydrogenase activity within the supernatant. Other laboratory parameters as CRP, PCT (normal value <0.05 μg/l), blood count and plasma creatinine levels were also measured at automated clinical chemistry analyses as part of the standard laboratory procedures at the Jena university hospital.

### Data analysis and statistics

Data were presented as median values and interquartile range (IQR). Statistical analysis was performed using GraphPad Prism (Version 5.01, GraphPad Software, Inc., USA). The Wilcoxon signed rank test was performed for dependent data sets within the same cohort. Continuous data were compared using the Mann–Whitney U test and the Kruskal-Wallis test with Dunn's Multiple Comparison Test. For correlation analyses Spearman correlation analyses (two tailed) were used. The Log-rank (Mantel-Cox) test was performed for survival analyses. The *P* value <0.05 was considered as significant. In correlation analyses results were quantified as low or weak (r≤0.35), modest or moderate (r = 0.36–0.67) and strong or high (r≥0.68–1.0) and confidence intervals are given. In healthy controls after exercise, analyzed parameters were normalized to the hematocrit before correlation analyses were performed.

## Supporting Information

S1 Table All clinical data from patients and healthy controls. (PDF)

S2 Table Data from exvivo whole blood stimulation analyses. (PDF)

S3 Table Survival curve data from mice treated with and without Lipocalin-2 (NGAL). (PDF)
