# Supplementary material for: Plasma Neutrophil Gelatinase-Associated Lipocalin Is Primarily Related to Inflammation during Sepsis: A Translational Approach
Source: PLoS One. 2015 Apr 20;10(4):e0124429. doi: 10.1371/journal.pone.0124429 (PMC4404058; doi:10.1371/journal.pone.0124429)
Supplement: S1 Table — (PDF) [file pone.0124429.s001.pdf]

Septic patients

Septic patients								
NGAL[ng/ml]	Crea[µmol/l]	EPI [ml/min/1,	IL-10 [pg/ml]	IL-6 [pg/ml]	IL-8 [pg/ml]	CRP [µg/ml]	ɔcytes *10³ [gı	PCT [µ/l]
1681,374	169	78,44898	50,31	3811,57	5373,16	413	16	32
192,8671	48	107,5779	8,61	193,74	78,43	295	24	0,8
244,6154	75	77,38144	22,6	42,23	155,6	57	19	1,2
310,3496	122	67,45956	13,44	51,71	89,26	163	14	0,4
863,0535	180	60,39148	39,52	370,66	161,17	150	15	4,4
141,1189	107	79,7867				88,5	17	0,1
381,6783	110	73,94015	18,15	78,67	148,38	49,5	9,8	1
1122,595	291	59,08829	0	226,76	240,54	225	23	8,3
64,1958	41	126,782	4,78	12,58	23,47	116	17	0,1
1927,176	299	54,85344	25,9	124,21	120,34	187	13	1,5
198,4615	105	73,27923	5,32	14,61	59,43	67	18	4,6
1017,252	214	68,4721	9,6	678,25	42,71	216	7,3	4,92
716,4885	131	66,71192	6,84	87,57	117,64	141	14,7	1,45
1082,901	131	68,87164	8,22	494,81	318,08	202	14,5	3,1
129,9301	77	77,25512	3,01	3,22	7,66	3,8	13,5	

Healthy controls

before							
NGAL[ng/ml]	Crea[µmol/l]	CRP [µg/ml]	EPI [ml/min/1,	IL-6 [pg/ml]	IL-10 [pg/ml]	IL-8 [pg/ml]	ɔcytes *10³ [gpt/µl]
36	52	1,70496	124,8979	0	4,42	7,58	10,2
28	57	1,88773	123,7627	0	0	5,84	4,7
47	43	1,49608	152,3959	0	5,95	6,79	4,2
42	61	1,46997	133,8627	0	5,41	7,95	4,9
32	60	1,88773	130,1237	5,5	0	0	4,4
34	63	1,91384	118,0832	0	0	8,3	6,9
24	71	1,8094	119,7313	5,71	0	10,88	3,1
36,46564	69	1,54047	123,7258	4,83	0	0	5,4

Healthy controls

after								
NGAL[ng/ml]	Crea[µmol/l]	CRP [µg/ml]	EPI [ml/min/1,	IL-6 [pg/ml]	IL-10 [pg/ml]	IL-8 [pg/ml]	ɔcytes *10³ [gı	Hct2
44	56	1,78329	121,8895	6,12	5,57	7,58	16,8	42,7
46	61	1,91384	121,0317	7,3	0	6,79	10,1	43,3
75	47	2,02611	146,9253	5,92	4,94	10,26	10,4	50,2
62	64	1,78329	131,2473	10,79	6,03	9,62	8,6	47,4
35	56	1,91384	133,8663	5,5	4,77	6,79	8	42,8
67	63	1,46997	118,0832	6,71	6,88	8,98	15	42,3
39	77	1,88773	115,805	8,69	0	0	7,1	46,4
72,93128	74	2,64491	120,219	5,81	4,42	8,3	10,2	49,5

Patients with rheumatic disease

before suppressing inflammation							
NGAL[ng/ml]	CRP [µg/ml]	ɔcytes *10³ [gı	EPI [ml/min/1,	Crea[µmol/l]	IL-8 [pg/ml]	IL-6 [pg/ml]	IL-10 [pg/ml]
46,28331	9,5	6	104,7675	63	23,84	0	0
98,17672	6,9	11,5	95,47071	69	16,24	38,32	31,4
158,2734	44,8	7,4	107,399	68	21,76	47,16	0
183,4532	22,9	12,4	145,9992	36	10,6	0	0
53,95683	139,1	9,3	122,1376	58	11,84	51,6	0
61,71108	17,6	9,5	98,43708	53	12,28	13,44	0
134,8921	44	6,3	103,865	48	10,2	0	0
134,6423	13,3	17,5	101,3613	64	9,76	0	0
41,36691	27,6	4,2	111,6049	63	11,44	0	0
88,35905	8,9	7,7	132,9711	62	8,08	0	0
98,17672	23,7	13,1	81,49863	76	8,92	0	0
172,6619	42	11,4	116,0513	35	29,68	14,84	0
174,4604	70,9	14,5	93,3568	74	25,16	57,84	0
77,13885	22,4	6,2	119,154	60	14,88	28,64	0
289,5684	32	9	87,75904	62	16,68	0	0
81,34642	13	8,3	109,2951	80	6,8	0	0
116,4095	15,6	10,1	74,70918	99	24,64	5,88	0
60,30856	14,6	8,9	106,2539	71	7,24	0	0
100,9818	6,2	10	123,1582	80	9,76	0	0
289,5684	116,1	12,1	78,45029	97	35,8	14,36	0
428,0576	135,2	7,6	120,5909	76	53,16	22,16	0
64,7482	21,8	6,7	107,2943	55	10,2	11,2	0
47,68583	17,4	5,4	108,1961	74	13,56	6,44	0

Patients with rheumatic disease

after suppressing inflammation							
NGAL[ng/ml]	CRP [µg/ml]	ɔcytes *10³ [gı	EPI [ml/min/1,	Crea[µmol/l]	IL-8 [pg/ml]	IL-6 [pg/ml]	IL-10 [pg/ml]
79,9439	1,9	7,5	103,4304	65	15,56	4,68	0
106,5919	22	6,4	97,86295	64	10,36	10,08	0
89,92805	66,3	8,5	106,7565	69	16,48	23,4	0
77,33813	3,1	6,5	122,7441	61	0	0	0
143,8849	2,4	5,7	118,0563	63	7,8	6,84	0
60,30856	13,1	8,6	97,24475	55	12,8	4,88	0
71,94244	23,4	6,5	99,91719	54	8,48	7	0
99,57924	1,9	12,6	100,8456	65	9,12	4,88	0
44,96403	1,9	3,4	107,5091	69	11,28	5,04	0
63,11361	1,9	5,5	128,7993	67	0	0	0
86,95652	5,5	10,1	83,34393	71	0	0	0
142,0863	23,9	12,4	93,72812	67	22,32	14,88	0
102,518	37,4	10,4	91,84436	77	21,64	19,12	0
35,06311	4,7	5,3	121,1818	57	0	0	0
77,33813	3,8	11,9	85,13209	68	0	0	0
77,13885	1,9	5,6	106,0942	86			
175,3156	8,1	11	73,5079	104			
77,13885	1,9	5,2	104,4619	74			
178,1206	6,4	8,5	121,9146	82			
167,2662	9,6	11,1	71,42635	129			
109,7122	44,2	4,2	121,9199	74			
52,15827	5,2	8,1	105,4358	58			
46,28331	3,5	5,7	110,0522	71			
