# Supplementary material for: Plasma Neutrophil Gelatinase-Associated Lipocalin Is Primarily Related to Inflammation during Sepsis: A Translational Approach
Source: PLoS One. 2015 Apr 20;10(4):e0124429. doi: 10.1371/journal.pone.0124429 (PMC4404058; doi:10.1371/journal.pone.0124429)
Supplement: S2 Table — (PDF) [file pone.0124429.s002.pdf]

Readout					NGAL [ng/ml]				
Stimulation	Control	LPS	IL-6	NGAL	LPS + NGAL	IL-6 + NGAL	NGAL(-S)	LPS+NGAL(-S)	IL6+NGAL(-S)
	162,809	285,2809	248,2023	1523,483	1867,303	1553,82	523,4832	867,3033	553,8202
	170,6742	398,764	204,382	2204,382	1621,236	1771,798	1204,382	621,236	771,7977
	151,573	368,427	196,5168	1987,528	2120,112	2085,281	987,5281	1120,112	1085,281
	160,5618	578,5393	297,6404	1947,079	1912,247	2172,921	947,0787	912,2472	1172,921

Readout		IL-6 [pg/ml]			IL-8 [pg/ml]	
Stimulation	Control	IL-6	NGAL	Control	IL-6	NGAL
	178,2	430,52	677,48	3214,52	3722,96	2313,32
	103,92	244,72	101	1775,68	2271,48	1712,04
	124,76	378,32	113,04	2313,32	2488,52	1775,68
	115,56	224,12	141,88	2355,92	1426,12	1759,56

Readout		IL-6 [pg/ml]			IL-8 [pg/ml]			IL-10 [pg/ml]			LDH [U/l]	
Stimulation	Control	LPS	LPS+NGAL	Control	LPS	LPS+NGAL	Control	LPS	LPS+NGAL	Control	LPS	LPS+NGAL
	178,2	54104,04	57617,96	3214,52	13828,4	20148,28	0		62,2	171	271	213
	103,92	55249,08	50819,12	1775,68	16507,2	11060,48	0	82,16	61,4	242	269	159
	124,76	59466,16	57617,96	2313,32	10642,36	8915,04	0	80,28	72,16	235	301	218
	115,56	58227,08	58227,08	2355,92	9485,44	9129,96	0	87,96	76,6	151	271	196
