# Supplementary material for: Plasma Neutrophil Gelatinase-Associated Lipocalin Is Primarily Related to Inflammation during Sepsis: A Translational Approach
Source: PLoS One. 2015 Apr 20;10(4):e0124429. doi: 10.1371/journal.pone.0124429 (PMC4404058; doi:10.1371/journal.pone.0124429)
Supplement: S3 Table — (PDF) [file pone.0124429.s003.pdf]

hours after Sepsis induction	Treatment (dead = 1, survival after 24h =0)	
hours	control	NGAL
12	1	
12	1	
12	1	
12	1	
12	1	
12	1	
15	1	
18	1	
15		1
15		1
15		1
18		1
18		1
21		1
24		0
24		0
24		0
